# Traveling by Bus Instead of Car on Urban Major Roads: Safety Benefits for Vehicle Occupants, Pedestrians, and Cyclists

**DOI:** 10.1007/s11524-017-0222-6

**Published:** 2018-03-02

**Authors:** Patrick Morency, Jillian Strauss, Félix Pépin, François Tessier, Jocelyn Grondines

**Affiliations:** 1 Université de Montréal Public Health Research Institute (IRSPUM) & Montréal Public Health Department, Montréal, Canada; 2 Department of Civil, Geological and Mining Engineering, École Polytechnique de Montréal, Montréal, Canada; 3 Agence Métropolitaine de Transport, Montréal, Canada; 4 Montréal Public Health Department, Montréal, Canada; 5 Société de transport de Montréal, Montreal, Canada

**Keywords:** Road injuries, Public transit and car safety, Pedestrian and cyclist safety, Injury rates and rate ratios

## Abstract

Some studies have estimated fatality and injury rates for bus occupants, but data was aggregated at the country level and made no distinction between bus types. Also, injured pedestrians and cyclists, as a result of bus travel, were overlooked. We compared injury rates for car and city bus occupants on specific urban major roads, as well as the cyclist and pedestrian injuries associated with car and bus travel. We selected ten bus routes along major urban arterials (in Montreal, Canada). Passenger-kilometers traveled were estimated from vehicle counts at intersections (2002–2010) and from bus passenger counts (2008). Police accident reports (2001–2010) provided injury data for all modes. Injury rates associated with car and bus travel were calculated for vehicle occupants, pedestrians, and cyclists. Injury rate ratios were also computed. The safety benefits of bus travel, defined as the number of vehicle occupant, cyclist, and pedestrian injuries saved, were estimated for each route. Overall, for all ten routes, the ratio between car and bus occupant injury rates is 3.7 (95% CI [3.4, 4.0]). The rates of pedestrian and cyclist injuries per hundred million passenger-kilometers are also significantly greater for car travel than that for bus travel: 4.1 (95% CI [3.5, 4.9]) times greater for pedestrian injuries; 5.3 (95% CI [3.8, 7.6]) times greater for cyclist injuries. Similar results were observed for fatally and severely injured vehicle occupants, cyclists, and pedestrians. At the route level, the safety benefits of bus travel increase with the difference in injury rate associated with car and bus travel but also with the amount of passenger-kilometers by bus. Results show that city bus is a safer mode than car, for vehicle occupants but also for cyclists and pedestrians traveling along these bus routes. The safety benefits of bus travel greatly vary across urban routes; this spatial variation is most likely linked to environmental factors. Understanding the safety benefits of public transit for specific transport routes is likely to provide valuable information for mobilizing city and transportation planners.

## Background

Road traffic is associated with several public health problems including urban air pollution, noise, physical inactivity, and injury. A modal shift towards public transit and active modes of transportation can reduce chronic diseases such as ischemic heart disease, cerebrovascular disease, and diabetes but, in urban settings, may be associated with an increase in the overall burden from road traffic injuries, through an increase in walking and pedestrian casualties [1, 2]. Understanding public transit’s contribution to road safety in urban settings is likely to provide valuable information for urban and transportation planners.

According to several studies, the rate of death is lower for travel on public transport than that in cars. For example, in the USA, fatality rate for car occupants were found to be 23 times higher than those for bus occupants, per 100 million person-trips [3]. Another study found fatality rate to be as high as 66 times greater for car occupants than those for bus occupants per passenger-mile traveled [4]. Similarly in Australia, car occupants have nine times greater rate of death than bus occupants, per hour traveled [5]. In Europe, car occupants have ten times greater rate of death compared to bus occupants and 20 times greater rate of death compared to train occupants, per kilometer traveled [6]. The non-fatal injury rate is also higher for car occupants compared to that for bus occupants: 4.3 times higher per kilometer traveled in Norway [7] and 5.0 times higher per person-trips in the USA [3]. These studies aggregated data for entire countries or groups of countries and therefore cannot describe the potential spatial variation across regions and contexts (e.g., urban versus rural). Furthermore, at the country level, no distinction is usually made between different types of busses (e.g., school bus, intercity, urban transit) [3, 6, 8], except for one study which only looked at fatality rates [9].

Two recent studies focused on urban areas and estimated that across major cities in the USA, an increase in the share of mass transit was associated with reduced motor vehicle fatalities, but the fatality rates were expressed as fatalities per city residents (as opposed to distance traveled) [10, 11]. However, in these city level studies, an ecological fallacy [12] cannot be ruled out: city level association between public transit use and lower fatality rate may not be observed at the route level. In other words, from studies which find that bus is safer than car overall at the city level, we cannot speculate about the bus routes. The city level results could be confounded by urban form and density, which are associated with public transit, distance traveled by car, and traffic fatality rate [9, 13–16]. Thus, in safer cities with more public transit, lower fatality rates may be attributable to other factors such as lower vehicle-miles traveled or lower speeds.

The rate of traffic injury and death can vary widely by road type and road network configuration [16]. Disaggregate analyses at the street level (e.g., intersections or roads) are necessary to estimate the effect of specific roadway characteristics on the likelihood of injury and death. Several Canadian studies developed collision prediction models to predict transit collisions at the zonal, intersection, and arterial levels accounting for transit network attributes as well as some geometry characteristics [17–19]. These studies however were limited to transit/bus crashes, and they did not compare the safety of car versus bus travel. A very limited number of studies considered specific bus preferential measures and road infrastructure, such as bus rapid transit [20–22] and transit signal priority [23], but these studies focused only on injuries associated with transit before and after the preferential treatments were applied.

Few studies considered non-motorized injured road users (pedestrians and cyclists) in the comparative analysis of public transit versus cars and other light vehicles. According to Litman, in the USA, the overall fatality rate (deaths per passenger-kilometer) associated with transit bus use—including deaths of bus occupants and other road users—was found to be much lower than for passenger car and light truck travel [24]. In the early 1980s, variation in London Transport bus and underground fares was found to be associated not only with bus and coach occupant casualties but also with pedestrian casualties [25]. Another country-level study found that busses were more likely to kill pedestrians than cars and light trucks [26], but it used vehicle-miles as the measure of exposure instead of passengers-miles or individual trips, it includes all types of bus travel, and it did not control for the volume of pedestrians, which might be greater on urban bus routes.

This work has two main objectives, to compare: (i) the rate of injury for car and city bus occupants on specific urban major roads and (ii) pedestrian and cyclist injuries associated with car and bus travel.

## Methods

The study environment is the island of Montreal (Canada), with a population of 1.8 million. Travel by bus and metro (around 1.2 million trips per day) are managed by the Société de transport de Montreal (STM).

### Traffic Routes

**Fig. 1:**
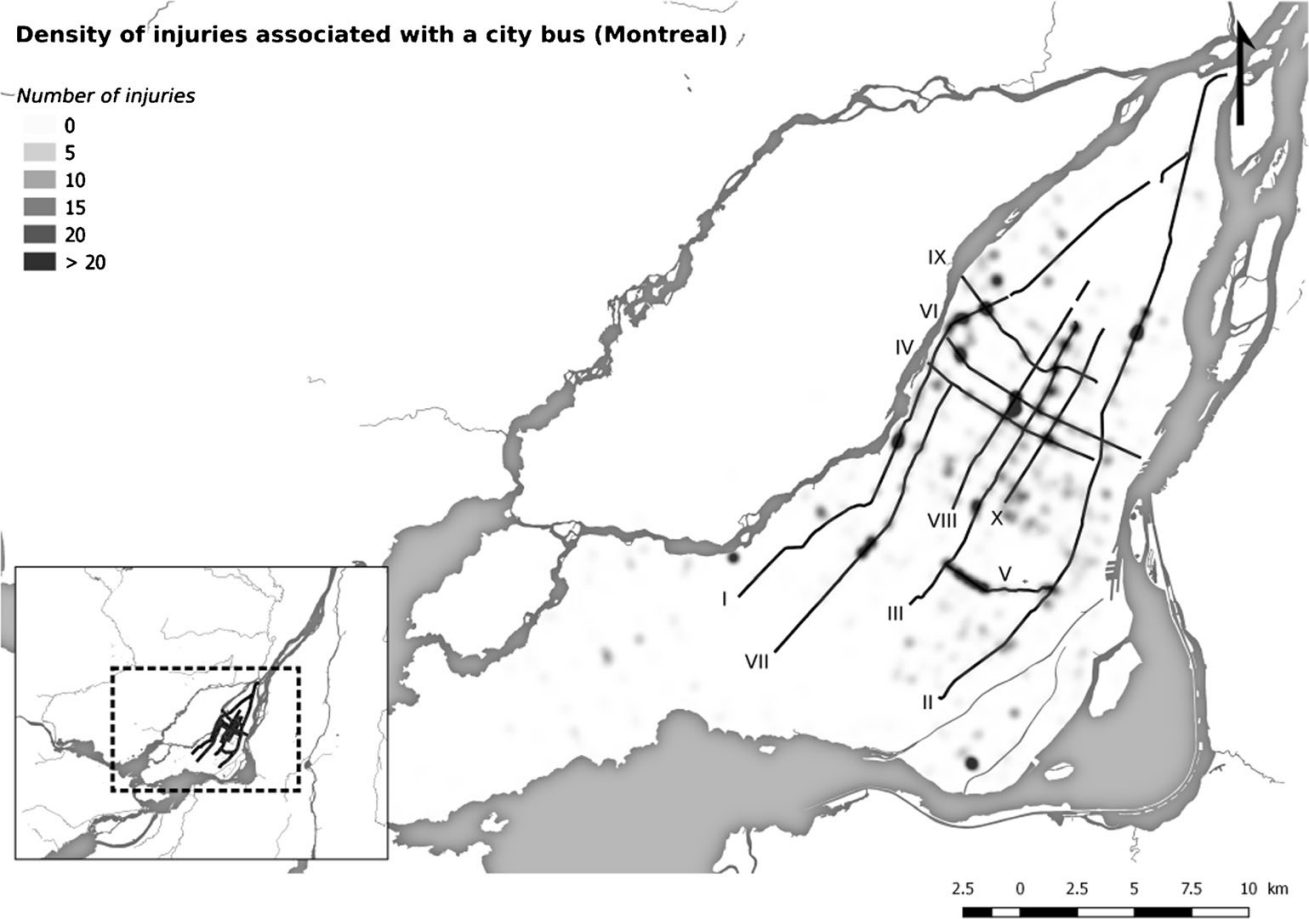
Density of injuries associated with a city bus (Montreal, Canada, 2007–2010)

Figure 1 shows the density of accidents involving a bus from which the STM professionals identified ten routes with the highest number of injuries involving a bus (2007–2010). These ten routes were selected for analysis and were divided into road sections, defined by entry and exit points of bus routes. Road sections which did not form part of a STM bus route were excluded.

For every STM bus line, the number of persons entering and exiting the bus was counted for all stops over a 24-h period, with automatic sensors installed on busses on a weekday, between September and December 2008 (data provided by STM). The daily number of bus passengers per road section was obtained by adding the number of passengers for each bus line traveling along the same road section. Travel by bus was converted to passengers per year by multiplying by 260 (representing the number of weekdays in a year).

Vehicle counts (2002–2010) at intersections along the ten selected routes were provided by the city of Montreal. The average daily number of vehicles was estimated for each vehicle direction (maximum 12 directions per intersection) using expansion factors (taking into account the time, day, and month of the vehicle count) traditionally used to produce the average annual daily traffic (AADT). The daily number of vehicles on road sections—between intersections with vehicle counts—was calculated by taking the sum of all vehicles entering the section and subtracting all the vehicles exiting at the next intersection. For two-way road sections, this procedure was applied to both directions of vehicular traffic. The daily number of car occupants on a road section was obtained by multiplying the number of vehicles on this section by the average number (1.23) of car occupants in Montréal (based on the 2008 Origin-Destination Survey).

**Fig. 2:**
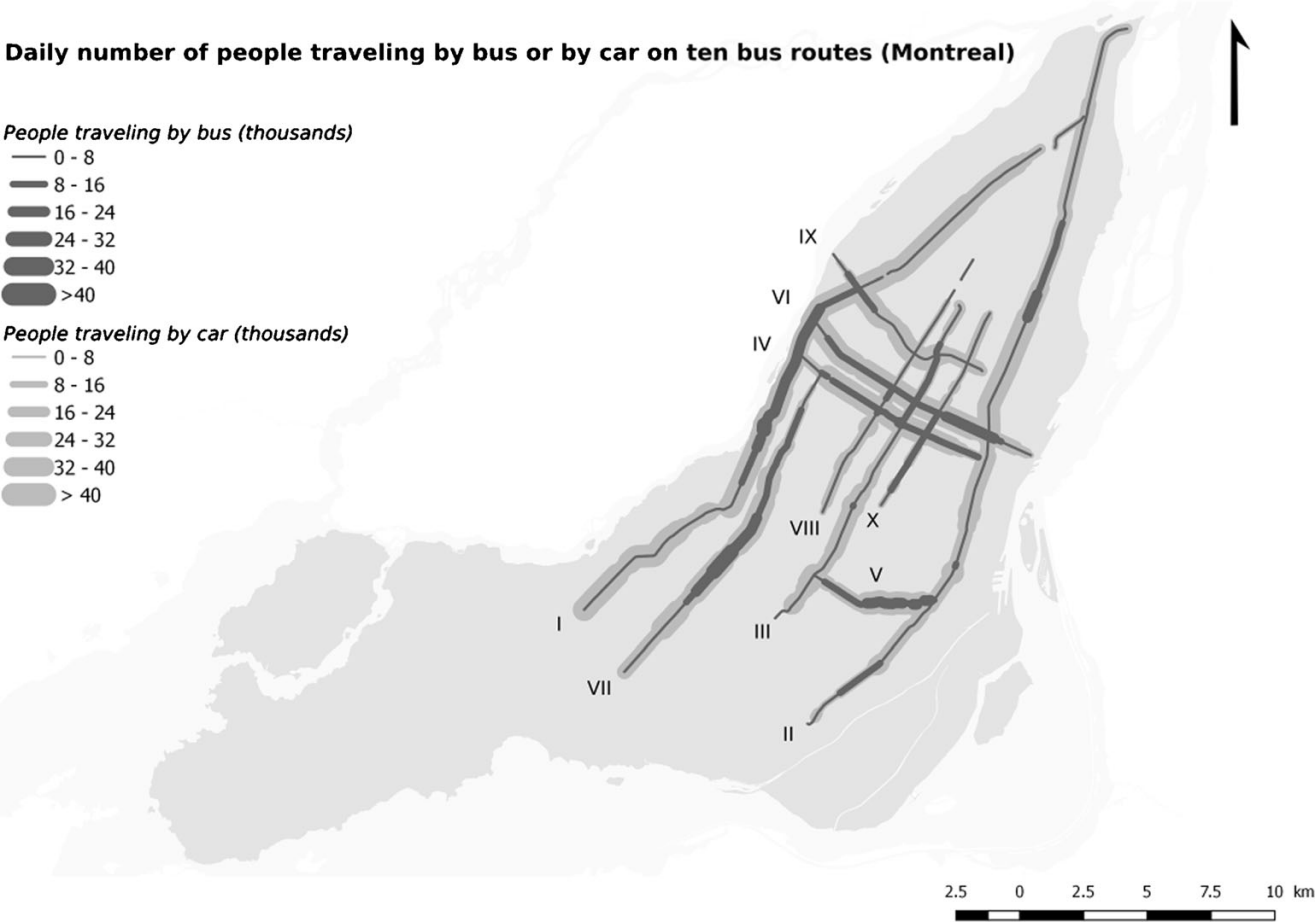
Daily number of people traveling by bus (2008) or car (2002–2010) on ten bus routes (Montreal, Canada)

For car and bus travel, passenger-kilometers were obtained by multiplying the number of people traveling on a road section by the length of the section. Then, the passenger-kilometers on each road section were summed for the ten routes studied (Fig. 2) and divided by one million to obtain car and bus travel in units of million passenger-kilometers per year.

### Collisions and Injuries

Weekday collision and injury data were extracted from police accident reports for 2001–2010 (Société de l’assurance automobile du Québec (SAAQ)) and divided by 10 years to obtain the average number of injuries per year. This source of data contains the date, collision location (address, name, and street type), road user class (driver, passenger, pedestrian, and cyclist), injury severity (minor, severe, and fatal), vehicle(s) involved in the accident, and vehicle occupied by the injured person(s) (weight, type, function). Collisions that occurred on highways, on a Saturday or Sunday, or having caused only property damage (without injuries) were excluded from this study.

The collision data was geo-coded (assigned geographic coordinates, x and y) using the police station number and the address (house number and street name) or, at intersections, using the name of the two intersecting streets as reported by the police.

Cars and light vehicles include cars, vans, and light trucks under 3000 kg and exclude heavier vehicles (greater than 3000 kg). Collisions involving city busses were identified by the SAAQ, using the vehicle type and the registration number of the vehicle (owner: STM). Thus, intercity busses and school busses were not included.

### Analysis

The annual number of injuries associated with car travel is compared to the annual number of injuries associated with bus travel and the following injury rates are computed: injury rates for city bus and car occupants (drivers and passengers), using Eq. 1a and 1b, respectively, and injury rates of pedestrians and cyclists injured by city bus or by car, using Eq. 2a and 2b, respectively.

1a $$ \mathrm{City}\ \mathrm{bus}\ \mathrm{occupant}\ \mathrm{injury}\ \mathrm{rate}=\frac{\mathrm{Number}\ \mathrm{of}\ \mathrm{city}\ \mathrm{bus}\ \mathrm{occupant}\mathrm{s}\ \mathrm{injured}\ }{\mathrm{Passenger}-\mathrm{kilometres}\ \mathrm{by}\ \mathrm{city}\ \mathrm{bus}} $$

1b $$ \mathrm{Car}\ \mathrm{occupant}\ \mathrm{injury}\ \mathrm{rate}=\frac{\mathrm{Number}\ \mathrm{of}\ \mathrm{car}\ \mathrm{occupant}\mathrm{s}\ \mathrm{injured}\ }{\mathrm{Passenger}-\mathrm{kilometres}\ \mathrm{by}\ \mathrm{car}} $$

2a $$ \mathrm{Rate}\ \mathrm{of}\ \mathrm{pedestrian}\ \left(\mathrm{or}\ \mathrm{cyclist}\right)\ \mathrm{injured}\ \mathrm{by}\ \mathrm{a}\ \mathrm{city}\ \mathrm{bus}=\frac{\mathrm{Number}\ \mathrm{of}\ \mathrm{pedestrian}\mathrm{s}\ \left(\mathrm{or}\ \mathrm{cyclist}\mathrm{s}\right)\ \mathrm{injured}\ \mathrm{by}\ \mathrm{a}\ \mathrm{city}\ \mathrm{bus}}{\mathrm{Passenger}-\mathrm{kilometres}\ \mathrm{by}\ \mathrm{city}\ \mathrm{bus}} $$

2b $$ \mathrm{Rate}\ \mathrm{of}\ \mathrm{pedestrian}\ \left(\mathrm{or}\ \mathrm{cyclist}\right)\ \mathrm{injured}\ \mathrm{by}\ \mathrm{a}\ \mathrm{car}=\frac{\mathrm{Number}\ \mathrm{of}\ \mathrm{pedestrian}\mathrm{s}\ \left(\mathrm{or}\ \mathrm{cyclist}\mathrm{s}\right)\ \mathrm{injured}\ \mathrm{by}\ \mathrm{a}\ \mathrm{car}}{\mathrm{Passenger}-\mathrm{kilometres}\ \mathrm{by}\ \mathrm{car}} $$

To compare the safety of travel by car and by city bus, rate ratios are calculated. The rate ratio between car and city bus occupant injuries is calculated as shown in Eq. 3a. The rate ratios used to compare pedestrian (or cyclist) injuries associated with car and city bus travel are calculated as shown in Eq. 3b. A rate ratio is also calculated to compare the total injury rates associated with car and city bus travel, where total refers to the injury rate for all road users. The rate ratio comparing the total injury rate therefore compares car occupants, pedestrians, and cyclists injured by a car to bus occupants, pedestrians, and cyclists injury by a city bus. Confidence intervals (CI, 95% thresholds) associated with the rate ratios of car to bus occupant injuries as well as pedestrian and cyclist injuries associated with car and bus travel are also calculated.

3a $$ \kern0.5em \mathrm{Ratio}\ \mathrm{of}\ \mathrm{vehicle}\ \mathrm{occupant}\ \mathrm{injury}\ \mathrm{rate}\mathrm{s}=\frac{\mathrm{Car}\ \mathrm{occupant}\ \mathrm{injury}\ \mathrm{rate}}{\mathrm{City}\ \mathrm{bus}\ \mathrm{occupant}\ \mathrm{injury}\ \mathrm{rate}\ } $$

3b $$ \mathrm{Ratio}\ \mathrm{of}\ \mathrm{pedestrian}\ \left(\mathrm{or}\ \mathrm{cyclist}\right)\ \mathrm{injury}\ \mathrm{rates}=\frac{\mathrm{Rate}\ \mathrm{of}\ \mathrm{pedestrian}\ \left(\mathrm{or}\ \mathrm{cyclist}\right)\ \mathrm{injuries}\ \mathrm{associated}\ \mathrm{with}\ \mathrm{car}}{\mathrm{Rate}\ \mathrm{of}\ \mathrm{pedestrian}\ \left(\mathrm{or}\ \mathrm{cyclist}\right)\ \mathrm{injuries}\ \mathrm{associated}\ \mathrm{with}\ \mathrm{city}\ \mathrm{bus}} $$

Separate analysis is performed for all injury severities (including minor, severe, and fatal) and for major injuries only (severe and fatal injuries). For all injury severities, the injury rates are estimated for each of the ten routes and overall for all ten routes. However, for major injuries, the injury rates are estimated overall for all ten routes since the number of major injuries associated with city busses is insufficient at the route level.

An additional step was carried out to estimate the safety benefits of current (2008) bus travel for each route. The benefit is defined as the number of vehicle occupant, cyclist, and pedestrian injuries saved per passenger-kilometer traveled by bus. This is measured by applying the injury rates estimated for passenger-kilometers traveled by car to the observed passenger-kilometers by bus. The number of injuries saved is then obtained by subtracting the number of injuries that were previously associated with bus travel from the computed injuries associated with the additional car travel. We also illustrate the association between the numbers of injuries saved as a function of the modal share of bus travel (bus passenger-kilometers/sum of bus and car passenger-kilometers).

## Results

### Kilometers Traveled and Injured Road Users

Overall, there were 4 times more passenger-kilometers traveled by car than by bus (1133 versus 257 million annual passenger-kilometers) and 16 times more injured car occupants (10,892) than bus occupants (668). Most pedestrians (95%) and cyclists (96%) were injured by a car.

Looking at major injuries only (excluding minor injuries), there were 28 times more injured car occupants (*n* = 278, including 19 deaths) than bus occupants (*n* = 10, no deaths). Cars were associated with 3 cyclist deaths and 42 pedestrian deaths while busses were associated with no cyclist deaths and 4 pedestrian deaths.

### Injury Rates

For all ten routes, the average injury rate per year for car occupants is 96.1 injured car drivers or passengers per hundred million passenger-kilometers (values range from 64.5 to 185.8 depending on the route) (Table 1(a)) whereas the average injury rate for bus occupants is 25.9 per hundred million passenger-kilometers (values range from 16.8 to 46.8 depending on the route) (Table l(b)).

**Table 1:** Annual injury rates associated with car and bus travel along ten bus routes (Montreal, Canada, 2001–2010)

(a) car travel—all injury severities					
Corridor	Million passenger-kilometers per year	Injury rate (per 100 million passenger-kilometers per year)			
		Car driver and occupant	Cyclist	Pedestrian	Total
I—Henri-Bourassa	292	66.4	2.6	9.5	78.5
II—Sherbrooke	247	95.0	12.1	20.4	127.5
III—Jean-Talon	91	152.8	11.2	42.4	206.4
IV—Saint-Michel	77	111.8	6.4	28.5	146.7
V—Côte-des-Neiges	45	80.3	9.3	35.7	125.3
VI—Pie-IX	112	99.1	6.3	21.5	126.9
VII—Côte-Vertu/Sauvé	118	64.5	3.2	16.2	83.9
VIII—Jarry	46	185.8	13.6	47.1	246.5
IX—Lacordaire	68	115.7	5.1	21.5	142.3
X—Beaubien	36	129.6	18.6	52.9	201.1
Overall	1133	96.1	7.4	22.4	125.9
(b) bus travel—all injury severities					
Corridor	Million passenger-kilometers per year	Injury rate (per 100 million passenger-kilometers per year)			
		Bus occupant	Cyclist	Pedestrian	Total
I—Henri-Bourassa	49	21.3	1.2	3.9	26.4
II—Sherbrooke	41	20.7	1.7	4.3	26.7
III—Jean-Talon	16	46.8	2.6	11.5	60.9
IV—Saint-Michel	24	25.8	2.5	6.2	34.5
V—Côte-des-Neiges	18	29.6	1.1	4.5	35.2
VI—Pie-IX	31	23.9	0.6	6.7	31.2
VII—Côte-Vertu/Sauvé	40	16.8	0.5	3.8	21.1
VIII—Jarry	10	40.1	1.9	13.4	55.4
IX—Lacordaire	13	38.8	3.0	3.8	45.6
X—Beaubien	14	37.7	0.7	4.9	43.3
Overall	257	25.9	1.4	5.4	32.7
(c) car and bus travel—severe and fatal injuries					
		Severe and fatal injury rate (per 100 million passenger-kilometers per year)			
		Vehicle occupant	Cyclist	Pedestrian	Total
Car travel	Severe injuries	2.29	0.34	1.87	4.5
	Fatal injuries	0.17	0.03	0.37	0.57
	All severe and fatal injuries	2.45	0.37	2.24	5.1
Bus travel	Severe injuries	0.39	0.04	0.43	0.86
	Fatal injuries	0.00	0.00	0.16	0.16
	All severe and fatal injuries	0.39	0.04	0.58	1.0

Major injury rates were calculated overall for all ten routes since the number of major injuries associated with city busses was insufficient for route level comparison

We quantified and compared pedestrian and cyclist injury rates associated with car and bus travel (Table 1). Looking at the same ten routes, the average rate of pedestrian injury associated with car travel is 22.4 injured pedestrians per hundred million car passenger-kilometers (values range from 9.5 to 52.9 depending on the route) (Table 1(a)) whereas the average rate associated with bus travel is 5.4 injured pedestrians per hundred million bus passenger-kilometers (values range from 3.8 to 13.4 depending on the route) (Table 1(b)). On average, the rate of cyclist injury associated with car travel is 7.4 injured cyclists per hundred million car passenger-kilometers (values range from 2.6 to 18.6 depending on the route) (Table 1(a)) whereas the rate associated with busses is 1.4 per hundred million bus passenger-kilometers (values range from 0.5 to 3.0 depending on the route) (Table 1(b)).

Overall, the total severe and fatal injury rate associated with car travel is 5.1 injured car occupants, pedestrians, and cyclists per hundred million car passenger-kilometers. Whereas, the total severe and fatal injury rate associated with bus travel is 1.0 injured bus occupants, pedestrians, and cyclists per hundred million bus passenger-kilometers (Table 1(c)).

**Fig. 3:**
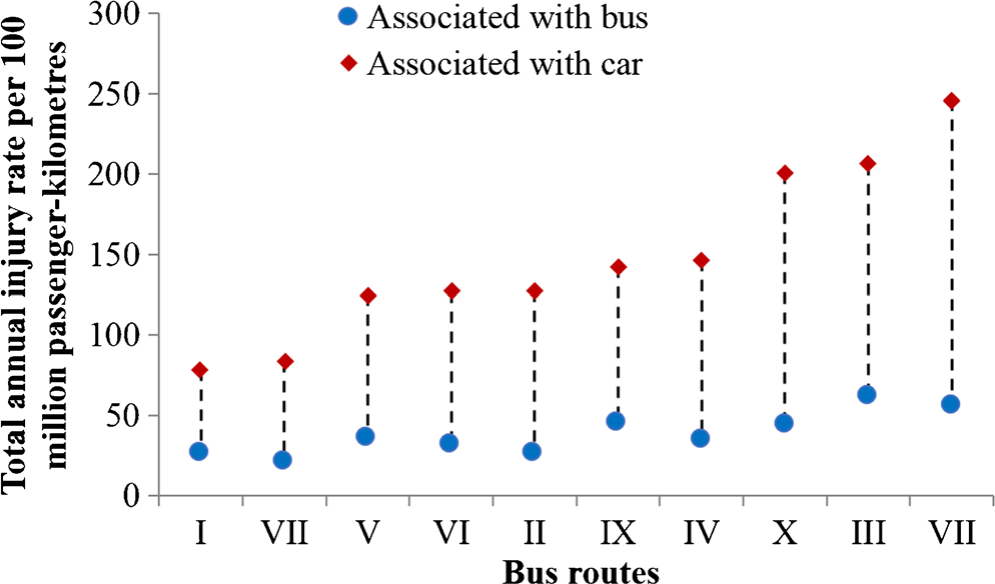
Difference in total annual injury rate associated with car and bus travel for ten routes (Montreal, Canada, 2001–2010). (Routes have been sorted from lowest to highest injury rate associated with car travel)

Injury rates associated with car and bus travel both vary greatly across the ten routes. Figure 3 shows the total annual injury rates associated with car and bus travel as well as the difference between these rates for each of the ten routes. The total annual injury rates associated with car and bus travel are highly correlated (*r* = 0.89). The benefits of bus travel—the number of injuries saved per passenger-kilometer—increase with the difference in injury rates associated with car and bus travel.

### Injury Rate Ratios

As another way to compare the injury rate for car and bus occupants, the rate ratios for each route, as well as the overall rate ratio across all ten routes were computed. Overall for all ten routes, the ratio between car and bus occupant injury rates is 3.7 (95% CI [3.4, 4.0]), emphasizing that the rate of injury is greater for car occupants than that for bus occupants per kilometer traveled. For each route, the injury rate ratio is significantly greater than one and, depending on the route, ranges from 2.7 to 4.6. The rate of fatally or severely injured vehicle occupants is 6 times greater for car occupants than for bus occupants (ratio = 6.3, 95% CI [3.4, 13.3]).

Overall, for all ten routes, the rate of pedestrian injury is 4.1 (95% CI [3.5, 4.9]) times greater for car passenger-kilometers than for bus passenger-kilometers. Depending on the route, the rate ratios range from 2.5 to 10.8. The rate of pedestrians fatally or severely injured is almost 4 times greater for car passenger-kilometers than that for bus passenger-kilometers (ratio = 3.9, 95% CI [2.3, 6.99]).

Overall, the rate of cyclist injury is 5.3 (95% CI [3.8, 7.6]) times greater for car passenger-kilometers than that of bus passenger-kilometers. Depending on the route, the rate ratios range from 1.7 to 26.7. The rate of cyclists fatally or severely injured is over 9 times greater for car passenger-kilometers than for bus passenger-kilometers (ratio = 9.3, 95% CI [1.6, 386.6]).

**Fig. 4:**
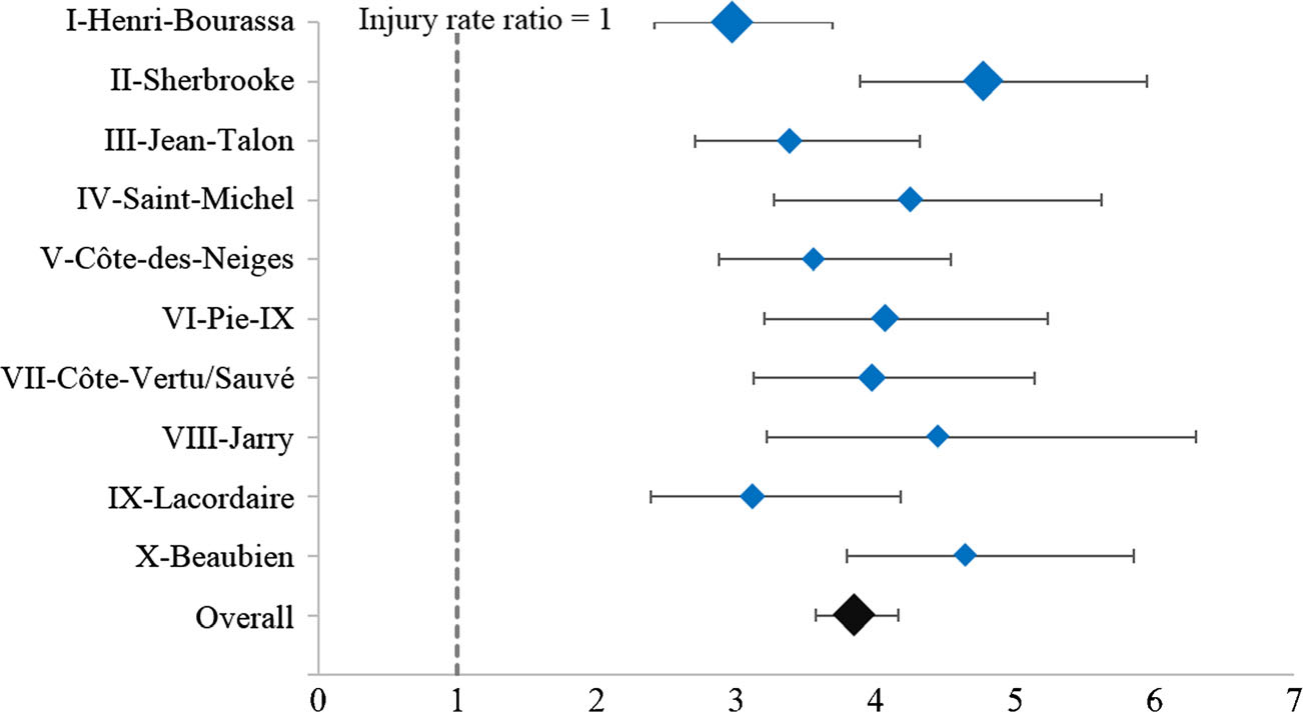
Total injury rate ratios associated with car and bus travel for ten bus routes (Montreal, Canada, 2001–2010). (Marker size is proportional to the total number of passenger-kilometers (car and bus))

The injury rates for vehicle occupants, cyclists, and pedestrians associated with car and bus travel were combined to obtain the total injury rate ratios on each of the ten routes and overall for all routes. Figure 4 shows these rate ratios. Overall for all ten routes, the ratio between injury rates associated with car and bus travel is 3.8 (95% CI [3.6, 4.1]) and for each route, the injury rate ratio ranges from 3.0 to 4.8.

### Safety Benefits of Bus Travel

**Fig. 5:**
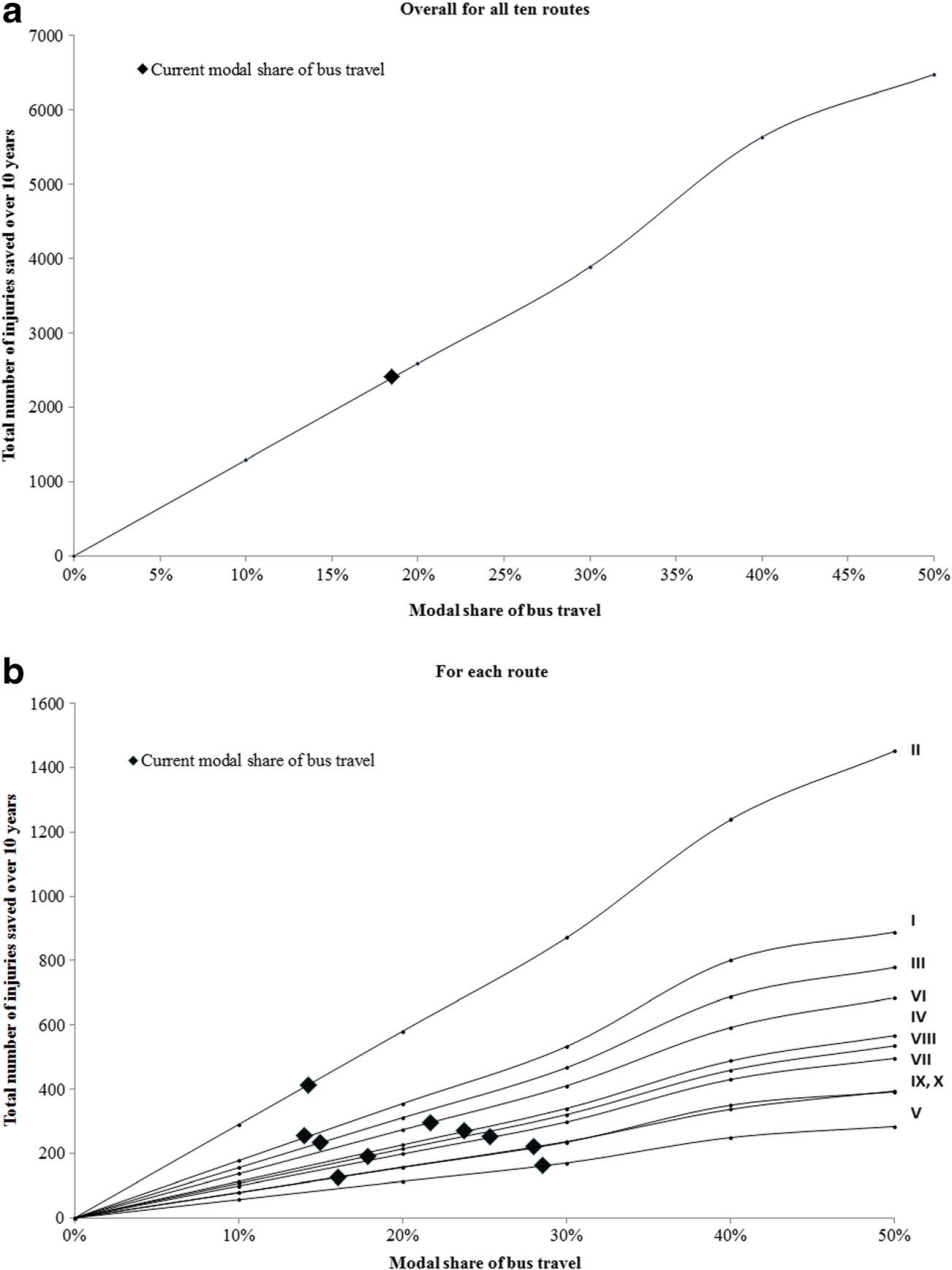
Total number of injuries saved over 10 years as a function of the modal share of bus travel (Montreal, Canada, 2001–2010)

To explore the safety benefits of current (2008) bus travel, we considered how the number of vehicle occupants, cyclists, and pedestrians injured would be affected if no travel was done by bus, by shifting all the passenger-kilometers by bus to car for each of the ten routes. Table 2 shows the difference in the number of injuries if all travel was done by car compared to the observed number of injuries on each route over 10 years. The number of vehicle occupants saved ranges from 91 to 308, the number of cyclists saved ranges from 3 to 43, and the number of pedestrians saved ranges from 23 to 69. The total number of injuries saved over 10 years for the ten routes ranges from 127 to 417, with an overall number of 2437 injuries saved resulting from bus travel on all ten routes. Of the 2437 injuries saved, 105 were severe or fatal (Table 2(b)). The safety benefits of current (2008) bus travel greatly vary across the ten routes. As shown in Fig. 5, the total number of injuries saved increases with the modal share of bus overall and on each route.

**Table 2:** Injuries saved by bus travel over 10 years along ten bus routes (Montreal, Canada, 2001–2010)

(a) all injury severities						
Corridor	Observed injuries	Injuries saved			Total injuries if no bus	
		Vehicle occupant^a^	Cyclist	Pedestrian	Number	Difference (%)
I—Henri-Bourassa	2419	223	7	28	2676	+11
II—Sherbrooke	3261	308	43	67	3678	+13
III—Jean-Talon	1977	166	13	48	2204	+11
IV—Saint-Michel	1210	207	9	53	1479	+22
V—Côte-des-Neiges	631	91	15	56	792	+26
VI—Pie-IX	1526	237	18	47	1827	+20
VII—Côte-Vertu/Sauvé	1072	190	11	50	1322	+23
VIII—Jarry	1199	153	12	35	1399	+17
IX—Lacordaire	1033	101	3	23	1160	+12
X—Beaubien	785	132	26	69	1011	+29
Total	15,113	1805	156	476	17,550	+16
(b) severe and fatal injuries						
	Observed injuries	Injuries saved			Total injuries if no bus	
		Vehicle occupant^a^	Cyclist	Pedestrian	Number	Difference (%)
Severe and fatal injuries	600	54	8	42	705	(+18)

^a^Vehicle occupant includes car drivers and passengers as well as bus occupants

## Discussion

This study achieved its objectives of comparing the rate of injury for car and city bus occupants as well as the injury rates for pedestrians and cyclists associated with car and bus travel on specific urban roads. The results reveal that, per kilometer traveled, bus travel is not only safer for vehicle occupants but also for pedestrians and cyclists traveling along these ten routes, although there is great variation across the routes.

Previous studies aggregated bus travel and injury data for whole regions or countries. This study provides injury rates per passenger-kilometer traveled by bus for each specific route. In Montreal, the routes with high injury rates associated with bus travel also have high injury rates associated with car travel (Fig. 3). This suggests that the same environmental factors (e.g., number of lanes, vehicle speed, and density of intersections) may be involved. The effect of road geometry on car occupant, pedestrian, and cyclist injuries has been extensively studied in the literature [16, 27–30], while very few studies have linked geometry to bus occupant injury occurrence [19]. A fairly recent review found mixed safety effects after the implementation of bus preferential measures, for example, a BRT (bus rapid transit), which was found to decrease injuries along some corridors but increase injuries along others and introduce new safety problems [31]. The design of public transit and general traffic lanes may both affect safety along transit corridors [20]. Disaggregate analyses at the route level are necessary to determine the effect of road geometry on injuries for all transportation modes.

By shifting all travel by bus to car, we estimated the current safety benefits of bus travel along each of the ten routes. Overall, bus travel saved 1805 vehicle occupants, 156 cyclists, and 476 pedestrians on ten urban bus routes (over 10 years). The absolute number of injuries saved by bus travel on each route varies with the amount of bus travel and with the difference in injury rates associated with car and bus travel. Our results show kilometers traveled by bus result in much fewer injured pedestrians than kilometers traveled by car. However, it is worth mentioning that in Canada’s large metropolitan areas, transit access points are concentrated at intersections of wide major roads with the greatest rate of crashes [19] and pedestrian injuries [32]. This reality also exists in cities like New York, where large arterials carry high volumes of car and bus traffic, at higher speeds than other streets and where pedestrian activity is greater [33]. Transit system main access points are often on busy arterials where pedestrians already have an elevated risk of injury compared to other modes, and with the improvement of public transit, more pedestrians are to be expected [20]. To reduce pedestrian injuries, a large reduction in car volume, area-wide implementation of traffic-calming measures, and safer pedestrian crossings are needed.

This study addressed some of the main shortcomings in the current literature. We only considered city busses and estimated car and bus passenger-kilometers along the ten chosen routes. To compare injury rates between bus and car travel, each route served as its own control therefore controlling, to some degree, for roadway environment, pedestrian, and cyclist volumes. No previous studies have compared the rate of injury for car and bus occupants at such a disaggregate level. Also, no studies have considered the rate of cyclist and pedestrian injuries associated with car and bus travel. One of the main obstacles to studying bus occupant injury occurrence is obtaining bus occupant exposure, in other words, knowing the number of people riding the bus along all segments making up its route. This study benefited from having automatic counters installed on each bus traveling along the ten routes.

Both car and bus injuries came from the same data source, but passenger-kilometers were estimated through different data sources. The passenger-kilometers are based on vehicle and bus occupant counts collected over one single day. This study did not take into account the daily, monthly, or annual variation in passenger-kilometers nor its evolution over the 10-year study period. We have no other data at the route level with which to validate our exposure data. An over-estimation of bus travel or an under-estimation of car travel would partially explain the observed results, but this study is likely over-estimating the car passenger-kilometers. For car travel, all types of vehicles were considered by the vehicle counts—even heavy trucks and busses—while only injuries associated with vehicles under 3000 kg were included. Regardless of these limitations, the injury rate ratio we found for car versus bus occupants is quite similar to the non-fatal injury rate ratio reported for the entire USA by Beck et al. [3].

To explore the variation in pedestrian and cyclist risk of injury (per pedestrian and per cyclist) across specific routes, estimates of pedestrian and cyclist activity would be required. Furthermore, road typology as well as geometric design and built environment is likely to vary throughout the route and therefore the injury rate is also likely to vary along the route. Future research will take into account the influence of geometric design of roads and include injuries associated with the walking portion of public transit trips.

## Conclusion

This study shows that city bus is a safer mode than car, for vehicle occupants but also for pedestrians and cyclists traveling along these bus routes. Although bus travel is safer on all specific routes, there is great variation in the safety benefits at the route level. The variation in injury rates and safety benefits of public transit is likely caused by road geometry and other environmental factors; disaggregate analyses at the route level are necessary to determine their effects. The results at the route level will provide vital information for cities to properly orient the implementation of environmental changes as preventative strategies to reduce the risk of injury for all road users.

## References

[1] Woodcock J, Edwards P, Tonne C, Armstrong BG, Ashiru O, Banister D, Beevers S, Chalabi Z, Chowdhury Z, Cohen A, Franco OH, Haines A, Hickman R, Lindsay G, Mittal I, Mohan D, Tiwari G, Woodward A, Roberts I (2009). Public health benefits of strategies to reduce greenhouse-gas emissions: urban land transport. Lancet.

[2] Stevenson M, Thompson J, de Sá TH, Ewing R, Mohan D, McClure R, Roberts I, Tiwari G, Giles-Corti B, Sun X, Wallace M, Woodcock J (2017). Land use, transport, and population health: estimating the health benefits of compact cities. Lancet.

[3] Beck LF, Dellinger AM, O’Neil ME (2007). Motor vehicle crash injury rates by mode of travel, United States: using exposure-based methods to quantify differences. Am J Epidemiol.

[4] Savage I (2013). Comparing the fatality risks in United States transportation across modes and over time. Res Transp Econ.

[5] Australian Transport Safety Bureau (ATSB). *Cross modal safety comparisons*. Australian Government: Australian Transport Safety Bureau; 2002.

[6] European Transport Safety Council (ETSC). *Transport safety performance in the EU—a statistical overview*.; 2003. http://etsc.eu/transport-safety-performance-in-the-eu-a-statistical-overview/. Accessed 20 Feb 2018.

[7] Elvik R (2009). The non-linearity of risk and the promotion of environmentally sustainable transport. Accid Anal Prev.

[8] Steer Davies Gleave. *What light rail can do for cities, a review of the evidence.* London (UK): Steer Davies Gleave; 2005.

[9] Litman T, Fitzroy S. Safe travels: evaluating mobility management traffic safety impacts. 2009.

[10] Stimpson JP, Wilson FA, Araz OM, Pagan JA (2014). Share of mass transit miles traveled and reduced motor vehicle fatalities in major cities of the United States. J Urban Health.

[11] Litman T (2014). A new transit safety narrative. J Public Transp.

[12] Diez-Roux AV (1998). Bringing context back into epidemiology: variables and fallacies in multilevel analysis. Am J Public Health.

[13] McAndrews C, Beyer K, Guse CE, Layde P. Are rural places less safe for motorists? Definitions of urban and rural to understand road safety disparities. *Inj Prev*. 2017:injuryprev-2016. 10.1136/injuryprev-2016-042139 28119341

[14] Ewing R, Cervero R (2010). Travel and the built environment: a meta-analysis. J Am Plan Assoc.

[15] Ewing R, Schieber RA, Zegeer CV (2003). Urban sprawl as a risk factor in motor vehicle occupant and pedestrian fatalities. Am J Public Health.

[16] Ewing R, Dumbaugh E (2009). The built environment and traffic safety: a review of empirical evidence. J Plan Lit.

[17] Quintero L, Sayed T, Wahba MM (2013). Safety models incorporating graph theory based transit indicators. Accid Anal Prev.

[18] Cheung C, Shalaby A, Persaud B, Hadayeghi A (2008). Models for safety analysis of road surface transit. Transp Res Rec J Transp Res Board.

[19] Shahla F, Shalaby A, Persaud B, Hadayeghi A (2009). Analysis of transit safety at signalized intersections in Toronto, Ontario, Canada. Transp Res Rec J Transp Res Board.

[20] Duduta N, Adriazola C, Hidalgo D, Lindau L, Jaffe R (2012). Understanding road safety impact of high-performance bus rapid transit and busway design features. Transp Res Rec J Transp Res Board.

[21] Tafur LE (2012). Impact of bus rapid transit systems on road safety. Built Environ.

[22] Tiwari G, Jain D (2012). Accessibility and safety indicators for all road users: case study Delhi BRT. J Transp Geogr.

[23] Goh K, Currie G, Sarvi M, Logan D (2013). Road safety benefits from bus priority: an empirical study. Transp Res Rec J Transp Res Board.

[24] Litman T. *Evaluating public transit benefits and costs*. Victoria, BC (Canada) : Victoria Transport Policy Institute; 2014.

[25] Allsop RE, Robertson SA. Road casualties in London in relation to public transport policy*. J Transp Econ Policy* 1994:61–82.

[26] Paulozzi LJ (2005). United States pedestrian fatality rates by vehicle type. Inj Prev.

[27] Harris MA, Reynolds CCO, Winters M, Cripton PA, Shen H, Chipman ML, Cusimano MD, Babul S, Brubacher JR, Friedman SM, Hunte G, Monro M, Vernich L, Teschke K (2013). Comparing the effects of infrastructure on bicycling injury at intersections and non-intersections using a case-crossover design. Inj Prev.

[28] Lovegrove GR, Sayed T (2006). Macro-level collision prediction models for evaluating neighbourhood traffic safety. Can J Civ Eng.

[29] Strauss J, Miranda-Moreno LF, Morency P (2014). Multimodal injury risk analysis of road users at signalized and non-signalized intersections. Accid Anal Prev.

[30] Greibe P (2003). Accident prediction models for urban roads. Accid Anal Prev.

[31] Vecino-Ortiz AI, Hyder AA (2015). Road safety effects of bus rapid transit (BRT) systems: a call for evidence. J Urban Health.

[32] Morency P, Archambault J, Cloutier M-S, Tremblay M, Plante *C.* Major urban road characteristics and injured pedestrians: a representative survey of intersections in Montréal, Quebec. *Can J Public Health* Vol 106, No 6 Sept. October 2015. http://journal.cpha.ca/index.php/cjph/article/view/4821. Accessed 20 Feb 2018. 10.17269/CJPH.106.4821 PMC6972380 26680430

[33] Viola R, Roe M, Shin H. The New York City pedestrian safety study and action plan. New York City, NY : New-York City Department of Transportation; 2010.

